# What Is Known about Theragnostic Strategies in Colorectal Cancer

**DOI:** 10.3390/biomedicines9020140

**Published:** 2021-02-01

**Authors:** Alessandro Parisi, Giampiero Porzio, Fanny Pulcini, Katia Cannita, Corrado Ficorella, Vincenzo Mattei, Simona Delle Monache

**Affiliations:** 1 Department of Life, Health and Environmental Sciences, University of L’Aquila, 67100 L’Aquila, Italy; alexparis@hotmail.it; 2 Medical Oncology Unit, St. Salvatore Hospital, 67100 L’Aquila, Italy; porzio.giampiero@gmail.com (G.P.); kcannita@gmail.com (K.C.); corrado.ficorella@univaq.it (C.F.); 3 Department of Biotechnology and Applied Clinical Sciences, University of L’Aquila, 67100 L’Aquila, Italy; fanny.pulcini@graduate.univaq.it; 4 Biomedicine and Advanced Technologies Rieti Center, Sabina Universitas, via Angelo Maria Ricci 35A, 02100 Rieti, Italy; v.mattei@sabinauniversitas.it

**Keywords:** CRC, liquid biopsy, CTC, ctDNA, mi-RNA, nc-RNA, gut microbiota

## Abstract

Despite the paradigmatic shift occurred in recent years for defined molecular subtypes in the metastatic setting treatment, colorectal cancer (CRC) still remains an incurable disease in most of the cases. Therefore, there is an urgent need for new tools and biomarkers for both early tumor diagnosis and to improve personalized treatment. Thus, liquid biopsy has emerged as a minimally invasive tool that is capable of detecting genomic alterations from primary or metastatic tumors, allowing the prognostic stratification of patients, the detection of the minimal residual disease after surgical or systemic treatments, the monitoring of therapeutic response, and the development of resistance, establishing an opportunity for early intervention before imaging detection or worsening of clinical symptoms. On the other hand, preclinical and clinical evidence demonstrated the role of gut microbiota dysbiosis in promoting inflammatory responses and cancer initiation. Altered gut microbiota is associated with resistance to chemo drugs and immune checkpoint inhibitors, whereas the use of microbe-targeted therapies including antibiotics, pre-probiotics, and fecal microbiota transplantation can restore response to anticancer drugs, promote immune response, and therefore support current treatment strategies in CRC. In this review, we aim to summarize preclinical and clinical evidence for the utilization of liquid biopsy and gut microbiota in CRC.

## 1. Introduction

Colorectal cancer (CRC) is the third leading cause of cancer-related death and morbidity worldwide according to the global cancer statistics (GLOBOCAN) presented in 2018. The 5-year survival rate ranges from 90% to 14% if CRC is diagnosed at a localized or metastatic stage, respectively, and approximately 25% of CRC patients present metastatic disease at diagnosis, while almost half of them will develop metastases [1].

If early diagnosis and treatment of CRC can significantly improve the cure rate, traditional biomarkers (Carcino Embryonic Antigen (CEA), Carbohydrate Antigen 19-9 (CA19-9), Fecal Occult Blood Test (FOBT)) as well as colon/sigmoidoscopy do not fully satisfy clinical needs in CRC screening due to their lack in sensitivity and specificity [2]. Furthermore, primary tumor resection is eventually associated to adjuvant chemotherapy with fluoropyrimidines with or without oxaliplatin according to TNM stage and pathological risk factors in early CRC [3], does not always seem sufficient to eliminate circulating tumor cells (CTCs) and other components involved in establishing pre-metastatic niche-promoting immune evasion and maintenance of stemness [4].

Circulating tumor DNA (ctDNA) and RNAs and non-coding RNAs (ncRNAs) released into the bloodstream via microvescicles or tumor cell lysis represent, together with CTCs, different sides of the same coin: liquid biopsy. Liquid biopsy has emerged as a promising minimally invasive tool for precision medicine due to its ability to provide multiple global snapshots of primary and metastatic tumors at different times and more representative images of the spatial and temporal tumor heterogeneity [5] compared to tissue biopsy. In fact, even though tissue biopsy remains the gold standard for the histopatological definition and the molecular stratification of tumors, it is often difficult to perform, especially in relapsed and metastatic settings, and it does not support intratumoral heterogeneity and clonal evolutions related to driver mutations, which may occur during tumor development or treatment.

Among other elements potentially involved in cancer initiation, development, recurrence, and metastasis, one that only recently received its due attention is the host microbiota—and for CRC, especially the gut microbiota. The host microbiota is composed of bacteria (≈99%), viruses, and mycetes, existing in a condition of eubosis with the human body conferring important benefits related to physical and mental health, and the development of the individual [6]. In turn, this dynamic balance is affected by host genetics, lifestyle [7], and dietary habits [8] and gut microbiota dysbiosis may play a role in promoting inflammatory responses and alterations of the immunosurveillance, which can led to cancer initiation and/or progression [9].

**Figure 1:**
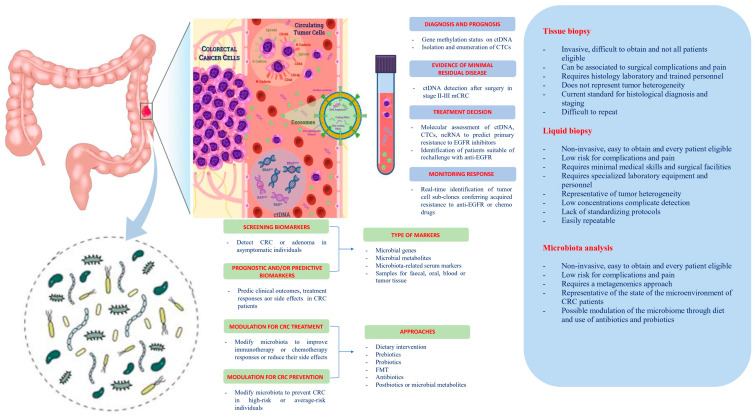
Potential clinical applications related to liquid biopsy and gut microbiota in colorectal cancer. Circulating tumor cells (CTCs), circulating tumor DNA (ctDNA), non-coding RNA (ncRNA), and exosomes are promising liquid biopsy markers for colorectal cancer with multiple potential advantages compared to tissue biopsy. CTCs from colorectal cancer (CRC) can be shed from the primary tumor into the bloodstream, which also contains ctDNA released from tumor tissue through apoptosis, necrosis, and secretion, as well as circulating normal DNA released from healthy tissue. NcRNAs (miRNAs and lncRNAs) encapsulated by exosomes can be actively secreted into the extracellular fluid by various types of cells in the tumor or passively released due to the apoptosis and necrosis of tumor cells and can eventually be found in the circulation. Besides liquid biopsy, several potential clinical applications for harnessing the gut microbiota in CRC include development of screening, prognostic and predictive biomarkers, and microbiota modulation for CRC prevention and treatment. FMT, fecal microbiota transplantation.

In this review, we summarize the state of the art regarding the potential role and the future perspectives of liquid biopsy and host microbiome as “theragnostic” tools in CRC (Figure 1).

## 2. Liquid Biopsy

The term liquid biopsy refers to procedures of isolation of cancer-derived components such as CTCs, exosomes, ctDNA, ncRNAs, and proteins from peripheral blood or other body fluids, and their genomic or proteomic evaluation [10]. Assessment of such elements via non-invasive and low-risk blood-based detection tests could improve CRC screening, diagnosis, staging, and predict relapse and metastasis [11,12] and be effective in monitoring residual disease and drug resistance in CRC patients receiving systemic treatment [13,14].

### 2.1. Circulating Tumor Cells (CTCs)

CTCs are tumor cells released into the bloodstream from the primary tumor or metastases [15], which could escape from immune recognition and drug treatment, and subsequently form a niche in other tissues, promoting tumor recurrence and metastasis [16].

#### 2.1.1. Screening and Early Diagnosis

Since counting CTCs reflects the patient’s tumor burden and the CTCs detection rate is positively correlated to the TNM stages, it is rather difficult and quite uncommon to detect CTCs in early-stage CRC, and therefore, their utility in CRC screening and early detection seems to be very poor [17]. However, a recent prospective study involving 667 patients (including healthy control subjects, patients with adenomas, and those with stage I–IV CRC) showed a significant association between CTC counts (performed using a novel CTC assay) and worsening disease status with respect to the adenoma-carcinoma sequence. Furthermore, the assay showed high specificity (86%) and sensitivity across all CRC stages (95%) and adenomatous lesions (79%) [18].

#### 2.1.2. Prognostic and Predictive Factor, Staging Tool and Guide for Systemic Treatment, Resistance Evaluation, and MRD Assessment

CTCs could potentially play a role as a prognostic marker, in monitoring treatment outcomes and follow-up, for modulating the intensity of systemic therapies and for detecting resistance against these. A meta-analysis of 15 studies including 3129 non-metastatic and metastatic CRC (non-mCRC and mCRC) patients showed significantly worse progression-free survival (PFS) and overall survival (OS) for CTC-positive with respect to CTC-negative CRC patients, regardless of sampling time (baseline or during treatment), detection methods (CellSearch, RT-PCR and others), and cut-off value of CTC (≥1, ≥2 and ≥3/7.5 mL blood), thus providing strong evidence for the presence of CTCs as an independent prognostic factor of poor survival [19]. A study conducted on 158 patients showed that rising of CTCs counts in 2 mL of peripheral blood (0 for healthy, 1 for benign, 5 for non-mCRC, and 36 for mCRC patients) was associated with tumor progression and poor prognosis at baseline. Notably, after 2 year follow-up on the non-mCRC patients, those who had ≥5 CTCs were eight times more likely to develop distant metastasis within one year after curable surgery than those who had <5 [20], therefore providing a support to the possible application of CTC detection during the follow-up of early CRC patients. Intensive first-line regimens with a triplet chemotherapy backbone plus the antiangiogenic bevacizumab provided better survival outcome if compared with doublet regimens, especially in *RAS*-*BRAF* mutated mCRC [21,22], paying the price of a major incidence of adverse events. Patient stratification by CTC detection could help modulate the intensity of the systemic treatment by reserving a more aggressive therapy to patients with a worse prognosis. In the randomized phase III VISNÚ-1 trial, a first-line systemic treatment with FOLFOXIRI (oxaliplatin, irinotecan, 5-fluorouracil (5-FU), and leucovorin) plus bevacizumab significantly improved PFS compared with FOLFOX (association of oxaliplatin, 5-FU, and leucovorin) plus bevacizumab in mCRC patients with ≥3 CTCs/7.5 mL blood at baseline [22]. In *RAS*-*BRAF* wild-type mCRC, a standard first-line regimen includes a doublet chemotherapy backbone in association with an anti-EGFR antibody (panitumumab or cetuximab), usually followed at disease progression by the alternative doublet regimen in association with an antiangiogenic drug [23,24]. As showed by a prospective study on 38 *RAS*-*BRAF* wild-type mCRC patients who received a third-line treatment with irinotecan and cetuximab, early CTC-negative and CTC status changes assessment during treatment were significantly associated with tumor response and better PFS and OS, predicting treatment failure in advance compared to imaging-based tools [25].

### 2.2. Circulating Tumor DNA (ctDNA)

Circulating tumor DNA (CtDNA) is a kind of double-stranded DNA, a fragment of cell-free DNA (cfDNA), that originates from active, apoptotic, necrotic, or circulating tumor cells. CtDNA retains epigenetic characteristics and harbors tumor-specific mutations detectable in the bloodstream and other body fluids [10,26]. Importantly, ctDNA half-life varies from several minutes to a few hours, and as for CTCs, its plasma levels depend on tumor load, ranging from 50% to 90% in non-metastatic and metastatic cancer patients, respectively [4,10,25,27]. Furthermore, healthy people and cancer patients can be distinguished according to the fragment length distribution pattern of cfDNA [26]. These data suggest that ctDNA analysis may represent a real-time tumor burden assessment.

#### 2.2.1. Screening and Early Diagnosis

Even if a recent meta-analysis concerning quantitative analysis of ctDNA for CRC screening, including 1258 CRC patients and 803 healthy individuals from 14 studies, concluded that the diagnostic accuracy of ctDNA has unsatisfactory sensitivity but acceptable specificity for CRC diagnosis [28], there is growing evidence that ctDNA detection could be used along with the traditional screening methods (i.e., colonscopy, FOBT, digital rectal examination, and serum tumor marker) to improve the diagnosis of early CRC [16,29]. In particular, ctDNA, especially when combined with carcinoembryonic antigen (CEA), showed higher diagnostic capacity (area under the ROC curve (AUC) 0.92, with 84% sensitivity and 88% specificity) [30]. Furthermore, epigenetic changes as DNA methylation and histone modifications are early events in carcinogenesis and clinical data that suggest that ctDNA methylation shows better sensitivity than traditional serum tumor markers in early-stage CRC [31,32]. Particularly, a meta-analysis of 25 studies assessing the diagnostic role of methylated Septin 9 (mSEPT9) promoter in ctDNA for CRC screening highlighted the efficacy of Epi proColon 2.0 with 2/3 algorithm (Epigenomics). A positive ratio of mSEPT9 was higher in advanced CRC stages (45%, 70%, 76%, 79% in I, III, III, and IV, respectively) and low-grade tumors (31%, 73% and 90% in high, moderate, and low grade, respectively), with a sensitivity, specificity, and AUC of 0.71, 0.92, and 0.88, respectively. Previous results confirmed the poor ability of mSEPT9 to identify precancerous lesions [31]. On the other hand, a recent prospective cohort study conducted on a high-risk population of 1493 individuals demonstrated that a particular single ctDNA methylation marker, cg10673833, could reach high sensitivity (89.7%) and specificity (86.8%) for the detection of CRC and precancerous lesions [32].

#### 2.2.2. Prognostic and Predictive Factor, Staging Tool, and Guide for Systemic Treatment, Resistance Evaluation, and MRD Assessment

A systematic review and metanalysis including 1076 mCRC patients treated with chemotherapy and/or targeted agents showed that lower baseline levels of cfDNA correlated with better OS [33]. A more recent systematic review and meta-analysis including 1779 non-mCRC and mCRC patients found that the presence or high concentration of ctDNA with KRAS mutation was associated with poor disease-free survival (DFS), PFS, and OS [34]. Moreover, as anti-EGFR therapy with cetuximab and panitumumab is approved for wild-type *RAS* mCRC and *KRAS* and *BRAF* are considered effective predictors of anti-EGFR therapy [35,36], ctDNA detection could represent an alternative tool for the selection of anti-EGFR treatment due to its correlation with *RAS* mutational status of tumor tissue [37]. In particular, *RAS* clones raised in blood during EGFR blockade decline after the withdrawal of anti-EGFR antibodies, therefore restoring the drug sensitivity of cancer cells and providing a rationale for anti-EGFR retreatment [37]. Moreover, ctDNA has a great potential to supplement Response Evaluation Criteria in Solid Tumors (RECIST) evaluation. As already discussed, ctDNA is strictly dependent by tumor load, and tumor burden can be monitored in real-time due to the short half-life of ctDNA [26,27]. Compared to radiological approaches, serial monitoring of ctDNA is able to track treatment response weeks to months earlier, allowing anticipating disease progression and modifying treatment consequently [37]. A prospective phase II clinical trial of cetuximab in *RAS* wild-type mCRC patients combined the sequential profiling of ctDNA and matched tissue biopsies with imaging and mathematical modeling of cancer evolution, showing that liquid biopsies were able to detect spatial and temporal heterogeneity of resistance to anti-EGFR monoclonal antibodies [38]. In another phase II trial that tested the multikinase inhibitor regorafenib in *RAS* mutated mCRC patients, combining dynamic contrast-enhanced (DCE), MRI, and ctDNA predicts the duration of antiangiogenic response to regorafenib, improving patient management with potential health and economic implications [39]. As for CTCs, ctDNA concentration is positively correlated with tumor size, resulting lower in stage I with respect to stage IV CRC patients [15,16,40]. As a result of the strong link between CTCs, ctDNA, primary tumor, and metastasis, it has been suggested to integrate the blood-based liquid biopsy into the actual TNM staging system, and the concept of “TNMB” (B as blood) has been proposed to improve the existing cancer staging system [2,4,41]. In this regard, the ability to optimize systemic treatments, especially in the adjuvant setting in stage II-III CRC patients, has been historically limited by the use of clinicopathologic characteristics, which are not always able to properly prognosticate the risk of recurrence [42], and by conventional surveillance modalities (CEA, computed tomography (CT), and colonoscopy), which are not perfectly able to identify MRD and early recurrence [2,4,11,12]. In a prospective cohort of 230 stage II CRC patients, 7.9% were postoperative ctDNA positive, 79% of whom relapsed, while disease relapse occurred only in 9.8% of ctDNA-negative patients. The presence of ctDNA after the completion of chemotherapy was also associated with worse recurrence-free survival [43]. In a recent prospective cohort of 130 stage I–III CRC patients, ctDNA was quantified pre- and postoperatively, and after adjuvant chemotherapy. CtDNA-positive patients after surgery, adjuvant chemotherapy, and during follow-up were respectively 7, 17, and 40 times more likely to relapse with respect to ctDNA-negative patients [44]. Some authors proposed that monitoring ctDNA levels every 3-6 months after surgery can be used to supplement serum markers, CT, endoscopy, and other conventional monitoring tools, emphasizing that positive ctDNA preceded radiological and clinical evidence of recurrence by a median of 3 months, even if 6% of patients with positive ctDNA never relapsed [45]. A great effort is ongoing to validate the clinical utility of ctDNA, particularly in the adjuvant setting of CRC (Table 1).

### 2.3. MicroRNAs (miRNAs) and Long Non-Coding RNAs (lncRNAs)

MicroRNAs (miRNAs) and long non-coding RNAs (lncRNAs) are ncRNAs molecules involved in the regulation of protein-coding gene expression through mRNA degradation and silencing or activating and repressing genes via a variety of mechanisms at both transcriptional and translational levels. Both classes of ncRNAs regulate multiple cellular processes such as growth, development, and differentiation showing to be crucial for cancer initiation, progression, and dissemination and can be found in serum or other body fluids bound to protein or lipid complexes, or more frequently inside extracellular vescicles (i.e., exosomes) [46]. Furthermore, these elements seem to be strongly associated with the development of drug resistance in CRC [47,48,49,50]. For these reasons, miRNA and lncRNAs could have potential application in diagnosis, prognosis, and treatment of CRC.

#### 2.3.1. Diagnosis and Prognosis

MiR-150 appears upregulated in CRC and its downregulation together with elevated Gli1 (glioma-associated oncogene homolog 1) expression seems to be involved in the process of epithelial–mesenchymal transition (EMT), which is a necessary step in promoting invasion and metastasis in CRC [51]. The results of a recent metanalysis suggest that miR-150 could be effective as a diagnostic biomarker for CRC patients, while no significant evidence was found concerning its prognostic role [52]. Mir-181 seems to be involved in multiple signaling pathways such as FOXO, PI3K-Akt, VEGF, HIF-1, mTOR, and cAMP, therefore representing a promising biomarker with potential predictive and prognostic significance in CRC [53]. MiR-21, miR-200a, miR-543, miR-32, miR92a, miR-26a, miR-1061, and miR-181a act as oncogenes downregulating the oncosuppressor PTEN (phosphatase and tensin homolog), which is a diagnostic factor for CRC patients, therefore representing potential targets for CRC therapy [54]. The upregulation of miR21, miR215, miR143-5p, and miR106a is associated with worse prognosis in stage II CRC patients [55]. A panel of miR-21, miR29a, and miR125b is able to carefully distinguish between early CRC and healthy controls (AUC = 0.827) [56]. Serum miR-203 upregulation seems to be related to worse prognosis (HR = 2.1) and higher risk of liver (OR = 6.2) or peritoneum (OR = 7.2) metastasis [57]. In a population of 400 CRC patients, a four-miRNA panel (miR-142-5p, miR-23a-3p, miR376c-3p, and miR271-3p) showed good diagnostic performance (AUC = 0.922), while a two-miRNA signature (miR-23a-3p and miR-376c-3p) proved to be a prognostic tool for 3-year OS (HR = 2.30) [58].

A study focusing on circulating serum exosomes showed that the levels of lncRNA HOTTIP could predict OS in CRC patients and discriminate between CRC and healthy controls (AUC = 0.75) [59].

A recent systematic review and meta-analysis of 111 articles including 13,103 gastrointestinal cancer patients (3123 with esophageal cancer, 4972 with gastric cancer, and 5008 with CRC) showed that 74 lncRNAs were closely associated with poor prognosis in gastrointestinal cancer, including 58 significantly upregulated and 16 significantly downregulated lncRNA expression, and with a strong interaction with miRNAs for 12 of these lncRNAs [60].

#### 2.3.2. Drug Resistance

Several oncogenic miRNAs can promote platinum and fluoropyrimidine resistance. Complex interactions between miRNAs (miR-181a-5p, miR-136, miR-363-3p, miR20b-5p, miR-218, miR-145, Let-7a, miR141) and lncRNAs (CRNDE, LUCAT1, MALAT1, GIHCG, CASC15, ANRIL, MEG3, CCAL) in the context of Wnt/β-catenin and MDM2-P53 signaling pathways are ultimately involved in oxaliplatin resistance [47]. Moreover, mir-153, miR19b-3p, miR-203, and miR-625-3p upregulation in the context of FOXO3a, SMAD4, and ATM pathways, respectively, is associated with oxaliplatin resistance [48]. The upregulation of LncRNA NEAT1 acts as an oncogene in CRC through the regulation of CPSF4 expression, sponging miR-150-5p. The upregulation of NEAT1 ultimately results in 5-FU resistance, suppressed apoptosis, and enhanced invasion of CRC [49]. A recent systematic review and meta-analysis of 39 studies including 2822 CRC patients consistently showed that multiple miRNAs (almost 60) could act as clinical predictors of chemoresistance and sensitivity for a combination of 14 drugs, including 5-FU and oxaliplatin. Particularly, 28 miRNAs were associated with chemosensitivity, 20 were associated with chemoresistance, 1 was associated with differential expression and radiosensitivity, while 10 were not associated with any impact on chemotherapy. These results outline the importance of almost 34 drug-regulatory pathways of chemoresistance and chemosensitivity in CRC that are potentially targetable [61].

## 3. Microbiota

The study of microbiota started several years ago, and multiple definitions have been conceived to explain its meaning [62]. In general, the terms “microbiota” and “microbiome” refer to the complex of organisms found within a specific environment and their genomic pool, respectively [63,64]. Thus, the human gut microbiota consists of a multitude of microorganisms colonizing the gut and existing in that complex state of dynamic equilibrium (i.e., eubiosis), which is made of reciprocal interactions and multiple networks between themselves and the host cells. This is an equilibrium with specific spatial and temporal characteristics, whose deregulation might lead to dysbiosis [63].

The human gut microbiota—with its thousands of different bacterial taxa, eucaryotic microbes, and virus together with the intestinal barrier—is a very selective and important filter for the well-being of the whole organism, and as a neuroendocrine structure today considered as a “second brain”, it is a component of the complex gut ecosystem [65,66]. The gastrointestinal microbiota varies according to the anatomical location and among individuals [11], and it plays different roles, from the supply of nutrients to the control of inflammation and carcinogenesis [63]. Commensal bacteria instruct the immune and physiological systems throughout life and are responsible for the presence of inflammatory and immune cells in the healthy intestine: the so-called “physiological” or “controlled” inflammation [67]. For this purpose, numerous evidence has demonstrated that a direct relationship between modification in the gut microbiota composition and some pathologies exist [68,69]. Among these diseases, obesity and metabolic alterations induced by some nutrients and diet, or autoimmune diseases such as type 1 diabetes and inflammatory bowel disease, are characterized by changes in the microbiome and gut dysbiosis [70].

### 3.1. Microbiota and Cancer

Gut microbiota emerged as a critical player also in the development of cancer. Several studies support the idea that a disturbance of the gut microbiota composition could lead to the onset of CRC [71]. Moreover, several studies reported a deep association between microbiota and CRC, demonstrating that microbiota dysbiosis can affect cancer susceptibility and progression through the modulation of several mechanisms such as inflammation, or inducing DNA damage, and producing metabolites involved in oncogenesis or tumor suppression [72]. For example, various bacterial pathogens are linked with the DNA damage response (DDR) pathway activation, which can be caused by both a direct effect of microbe produced genotoxins or an indirect effect of ROS produced in response to an excessive activation of immune cells stimulated by certain microbes or their metabolic end-products [73,74]. 

In particular, fecal metagenomic samples from CRC patients identified a CRC-enriched microbiota including *Enterobacteriaceae* [75], *Escherichia coli* [76,77], Enterotoxigenic *Bacteroides fragilis* (ETBF) [78], and *Fusobacterium nucleatum (Fn)* [79]. These bacteria seem to act as “pro-oncogenic” agents in different ways: promoting inflammation, impairing antitumor activity, inducing DNA damage, and tumor cell proliferation via the activation of β-catenin and other oncogenic pathways [75]. Several studies reported an association between an abundance of *Fusobacterium nucleatum*, carcinogenetic risk factors, and gene mutations in CRC [80]. In addition, a high abundance of *Fusobacterium nucleatum* was associated with CIMP status, wild-type p53, and MSI in colon tumor tissue [81]. 

On the other hand, the *Firmicutes* phylum (particularly the *Ruminococcaceae* and *Lachnospiraceae* families) [82] as well as *Bifidobacteria, Lactobacilli* [83,84] and non-enterotoxigenic *Bacteroides fragilis* (NTBF) [84] are substantially underrepresented in CRC patients [85] and have shown “anti-oncogenic” activities, such as a reduction of pro-inflammatory citokines, enhancement of antitumor immunity, epithelial cell renewal, regulation of intestinal barrier integrity, and short-chain fatty acid (SCFA) production [82,83,84,85,86]. SCFAs, by modulating histone deacetylase inhibitory activity, promote the accumulation and differentiation of Treg cells controlling tumor progression [86].

Similarly, a deeper review on the role of gut microbiota in the carcinogenesis of humans and animals observed that some bacteria appeared often augmented (including Fusobacteria, *Alistipes*, Porphyromonadaceae, Coriobacteridae, Staphylococcaceae, *Akkermansia* spp., and Methanobacteriales), whereas others decreased in CRC (*Bifidobacterium, Lactobacillus, Ruminococcus, Faecalibacterium* spp., *Roseburia*, and *Treponema* [87]. In addition, some microbial metabolites (such as nitrogenous compounds) were consistently elevated, whereas others (such as butyrate) were decreased throughout colonic carcinogenesis [87].

### 3.2. Signaling Pathways Activated in Microbiota and Cancer

The gut microenvironment homeostasis requires an intricate balance between cell proliferation, differentiation, and apoptosis processes in which several regulatory pathways are involved such as the Wnt, Notch, BMP, and Hedgehog signaling pathways [88,89]. Deregulation of these main signaling pathways can potentially determine a disruption of intestinal homeostasis and contribute to CRC development. For example, the Wnt/β-catenin signaling pathway is supposed to be closely connected with cancer biology [90]. In particular, the *adenomatous polyposis coli (APC)* gene truncating mutations that stabilize β-catenin are highly prevalent in CRC, making *APC* one of the most mutated genes in human cancers [91].

Among the canonical and non-canonical Wnt signaling pathways, the first is certainly the most critical for its function as regulator of the transcriptional co-activator β-catenin, in turn regulating inflammatory, proliferative, and differentiation pathways [92,93]. As reported in several studies, the Wnt pathway has been frequently considered together with the *RAS* pathway one of the major drivers of CSC expansion [93].

The gut microbiome can be the trigger of the (EMT), a transition taking place through the involvement of WNT and TGF-β signaling, as previously reported, causing the invasion and metastasis of CRC cells [94].

Recently, preclinical evidence demonstrated that defects in the colon barrier integrity associated with dysbiosis and with an increased expression of several inflammatory factors such as IL-17, *Cxcl2, Tnf-α,* and IL-1 can be responsible for the development of benign (e.g., hyperplastic polyp), pre-malignant (e.g., tubular adenoma), or malignant (e.g., colorectal adenocarcinoma) neoformations [71,95]. 

Taken together, these data suggest that alterations of gene expression or modifications of microbiota composition can trigger the development of cancer involving the deregulation of proliferative and inflammatory signaling pathways even though a clear cause–effect relationship between microbiota composition and changes in gene expression have not been well elucidated. 

In conclusion, not only a genetic but also an epigenetic role has been highlighted in CRC progression and metastatization, as recently reported by Wu et al. [96].

### 3.3. Microbiota and Efficacy of Anticancer Agents

An emergent approach is taking into consideration the influence of the microbiota on the activity and efficacy of chemotherapy and immunotherapy drugs. For example, the hypothesis that gut microbiota can be strictly related to the pharmacological effects of chemotherapy agents, such as 5-FU, is supported by a pioneer study conducted with a CRC mouse model and high-throughput sequencing. The authors compared the tumor size and profiled the gut microbiota of mice treated with 5-FU, combined with probiotics or ABX (an antibiotic cocktail of antibiotics), demonstrating the importance of pre-existing gut microbiota communities in the host response to 5-FU treatment. In particular, they found that antibiotics-induced dysbiosis during CRC treatment determined a dramatic increase of Proteobacteria, which may interact with the host inducing systemic inflammation and abolishing the therapeutic efficacy of the drug [97].

Regarding human studies, Zhang and colleagues investigated the relationship between *Fn* infection and efficacy of a systemic treatment with 5-FU in 94 CRC patients. They initially hypothesized a mechanism of reduced chemo-sensitivity of CRC cells to 5-FU linked to the upregulation of BIRC3, which is a member of the inhibitor of apoptosis proteins (IAPs). Next, they demonstrated that *Fn*-induced BIRC3 expression could be mediated by the TLR4/NFkB pathway. Indeed, other scientists had recently reported that *Fn* may mediate chemoresistance by activating the autophagic pathway in CRC [98].

Immunotherapy has revolutionized cancer treatment, and immune checkpoint inhibitors (ICIs) are now a standard of care in microsatellite-instable (MSI) CRC patients [99].

Recently, Lang et al. in their study showed that ileal microbiota can orchestrate the immunogenic cell death of ileal intestinal epithelial cells (IECs). They registered an accumulation of follicular T-helper (TFH) cells in CRC patients and mice and the suppression of IEC apoptosis. This effect could be linked to the impairment of the immunosurveillance mechanisms by chemotherapy directed against CRC in mice [100]. Protective immune responses in the ileum were associated with the colonization of specific bacteria such as *Bacteroides fragilis* and *Erysipelotrichaceae* that stimulate the production of programmed cell death (PD-1) molecules +TFH by secretion of interleukin 1R1 and interleukin 12. Moreover, the demonstration of apoptosis in the ileum can be considered a prognostic factor for CRC patients [100].

As for the relationship between bacteria species infection and efficacy of treatments, it has been postulated that the richness and diversity of species could be influenced by the different stages of gastric carcinogenesis and progression. In particular, more relevant changes seem to occur at the stage of precancerous lesions of gastric carcinoma (PLGC), suggesting that it is a turning point during GC progression. Moreover, the depletion of some bacteria such as *Akkermansia* and an enrichment of pathogenic bacteria such as *Escherichia Shigella* can overlap with the tumor progression stage [100]. 

Moreover, researchers have reported a reduction in the efficacy of immunotherapy regimens in metastatic renal cell carcinoma (mRCC) patients when treated with antibiotic drugs. In particular, worse clinical outcomes in terms of PFS and OS were found in mRCC patients who received antibiotics within four weeks of treatment initiation with respect to non-users [101].

### 3.4. Recent Advances in Metagenomics Technology for Diagnosis and Prognosis

Currently, the study of microbiomes, also named metagenomics, is based on two main approaches, which consider different aspects of the microbial community in a given environment. The structural metagenomics approach takes into consideration the structure, composition, and dynamics in a specific ecosystem of the uncultivated microbial population. Instead, functional genomics aims to study a specific gene coding for a function of interest. This approach requires the generation of expression libraries with thousands of metagenomics clones and its subsequent screening [102].

Metagenomics, investigating the wide populations of microbial communities and analyzing all the DNA present within a sample, can provide comprehensive and useful data regarding the state of the microenvironment of CRC patients. In metagenomics, datasets acquired from recent studies of the taxonomic clades related to CRC have been discovered [103]. 

Moreover, Meyerson et al. by using whole-genome sequences established the configuration of microbiota in healthy and CRC patients [104]. By the way, thanks to the multi-omics approach based on the plethora of recent technologies including genomics, transcriptomics, proteomics, and metabolomics respectively able to analyze DNA markers, RNA transcript, protein, and metabolites produced inside the colon, researchers have a remarkable opportunity for the discovery of novel prognostic, diagnostic, and therapeutic biomarkers [104], even though the question of whether the microbiota and its metabolites could be considered replicable and useful biomarkers across cohorts and populations remains unclear. So, the aim of this interesting approach tries to examine the differences in patients and healthy individuals for identifying biomarker patterns to work toward a personalized medicine therapeutic approach [105].

For example, in a large cohort study conducted on 616 participants undergoing colonoscopy, the presence of distinct patterns of the microbiome in cases of multiple polypoid adenomas has been demonstrated. *Fn* appeared significantly elevated from intramucosal carcinoma to more advanced stages. Moreover, *Atopobium parvulum* and *Actinomyces odontolyticus*, which co-occurred in intramucosal carcinomas, were significantly increased only in multiple polypoid adenomas and/or intramucosal carcinomas. In addition, metabolome analyses indicated a significant increase of metabolites such as branched-chain amino acids and bile acids in intramucosal carcinomas. Futhermore, the authors suggested that the shift in the microbiome and metabolome seemed to occur from the very early stages of CRC development, confirming the potential diagnostic and etiological role of multi-omics data. Therefore, the authors proposed metagenomic and metabolomic markers to discriminate cases of intramucosal carcinoma from the healthy controls, highlighting the possible etiological and diagnostic importance of large-cohort multi-omics data [106]. Indeed, the application of metagenomics to explore the gut microbiota profile has also been prospectively investigated in 60 CRC patients and 30 healthy controls. This study revealed the importance of data from the gut microbiome in association with known clinical risk factors of CRC to discriminate between adenoma and carcinoma clinical groups [107]. On the other hand, a similar conclusion has been reported by a European study based on fecal samples metagenomic sequencing and taxonomic classification of a mixed group of CRC, adenomas patients, and healthy subjects. Indeed, this study indicated that observed gene pool differences may reveal tumor-related host–microbe interactions [108].

An emerging approach to study the intersection of the gut–microbial communities and human health is based on the study of microbe-derived extracellular vesicles (EVs). EVs, separated into three different types, outer membrane vesicles (OMVs), shedding vesicles, and apoptotic bodies, are composed of different macromolecules including lipids, proteins, nucleic acids, and metabolites [109,110].

For example, via metagenomic and metabolomics analysis of gut EVs of CRC and healthy subjects, Kim et al. found an alteration of compositional bacteria and metabolites profile in CRC patients, suggesting a potential diagnostic role of EVs metabolites profiles in the identification of cooperation between microbiome and cancer development [109].

### 3.5. Organoids Engineering

Organoid engineering has become an important tool for cancer assessment but also in modeling host–microbe interactions. New insights are rapidly being gained on the role of the microbiome in CRC development, and it is clear that CRC patients have an altered gut microbe population compared to healthy ones. However, whether they play a direct or indirect role in cancer development is a topic of great discussion [111].

Research suggests a key role for microbes in developing an inflammatory environment in which cancer cells can grow; they can also influence cancer development by producing metabolites that influence the host metabolism [112].

From a practical point of view, microbes can be administered to cell culture media, allow basolateral exposure, or be microinjected into the lumen of the organoid to faithfully reproduce the microbial activity [113].

For example, Pleguezuelos-Manzano et al. focused on the abundance, in stool samples of CRC patients, of some bacteria including *E. coli* and pks + *E. coli*, which are capable of producing the genotoxin colibactin. This toxin has been shown to damage DNA and create a non-physiological base pairing in epithelial cells [77].

By the use of organoids constituted with *E. coli* pKs + obtained from the colon of CRC patients co-cultured with the epithelial cells, these researchers reproduced in vivo the intestinal situation and demonstrated that exposure to *E. coli* pKs + would appear to be a risk factor in the development of CRC [114]. Therefore, in conclusion, the specificity of colibactin-induced mutations supports the need for further investigations relating to its link with cellular DNA as well as representing a valid support in the identification of a preventive biomarker [77].

New knowledge is also rapidly gaining in the field of “nutrition and gut microbiota”. Several studies have established that after the ingestion of phytochemicals and fibers, the intestinal microbiota initiates complex catabolism that releases important metabolites of the intestinal microbiome (GMMs). Moreover, thanks to the use of organoids derived from colorectal lesions, the impact of diet and metabolites on tumorigenesis has been also investigated [115].

Recently, Toden and colleagues identified evidence that metabolites produced by the microbial catabolism of flavan-3-ols in the distal gastrointestinal tract could induce programmed cell death, inhibiting cancer and promoting gut health [116]. They used intestinal organoids as a preclinical model system and noted that flavan-3-ols suppressed the formation and growth of both intestinal organoids—those derived from APCM in mouse models and those from human CRC tumors—by inhibiting the cell cycle and inducing apoptosis. The gene expression profile revealed the suppression of survival and self-renewal pathways in organoids treated with flavan-3-ols. Flavan-3-ols is a commercial grape seed extract, consisting of monomers, dimers, and trimers. These compounds include proanthocyanidins (PACs); they can reach the distal gastrointestinal tract almost intact and are effectively transformed into low molecular weight phenolic compounds by the colonic microbiota [117,118,119]. The flavan-3-ols monomers, dimers, and trimers that reach the colon become available for the gut microbiota. Then, microbial catabolism begins, producing hydroxy-phenyl-γ-valerolactones (PVLs) and, to a lesser extent, their derivative hydroxy-phenylvaleric acids (PVAs), with only a small percentage of non-metabolized PACs remaining [120].

### 3.6. Therapeutic Use of Antibiotics, Probiotics, and Fecal Microbiota Transplantation

Several approaches, which include dietary interventions, antibiotic treatments, pre- and probiotics, and fecal microbiota transplantation (FMT), have been explored to modulate gut microbiota composition, including its physiology and metabolites involved in CRC occurrence, progression, or drug resistance. 

Diet plays a significant role in the modulation of the microbiome. A normal gut microbiota depends upon the fermentation of the indigestible fiber component of our diet for its energy supplement. The symbiotic gut microbiota ferments dietary fibers into short-chain fatty acids (SCFAs) such as propionate, acetate and, most importantly, butyrate [67]. In a prospective cohort study, a diet rich in whole grains and dietary fiber was associated with a lower risk to develop *F. nucleatum*-positive CRC but not *F. nucleatum*-negative CRC, supporting a potential role for intestinal microbiota in mediating the association between diet and colorectal neoplasms [121]. As no clear guideline regarding the type of nutrition and cancer incidence has been established, different forms of reduced caloric intake, such as fasting, demonstrated a wide range of beneficial effects in cancer prevention and anticancer drug efficacy [122], at least in part mediated by gut microbiota. Indeed, every-other-day fasting leads to an increase in fermentation products such as acetate and lactate altering gut microbiota composition, with enriched levels of Firmicutes, the production of SCFAs, and reduction in Bacteroides, Actinobacteria, and Tenericutes [123]. Since tumors are not able to metabolize ketone bodies due to deficiencies in key mitochondrial enzymes, a ketogenic diet with low-carbohydrate and high-fat intake, mimicking the metabolic state of fasting by inducing a physiological increase in acetoacetate and beta-hydroxybutyrate, might be a reliable therapeutic strategy to inhibit cancer progression [124]. Omega-3 polyunsatured fatty acids (PUFAs) are widely used as nutritional supplements and multiple benefits have been claimed, included anticancer activity. PUFAs seem to increase “anti-oncogenic” bacteria, including *Bifidobacterium* and *Lactobacillus* other than SCFA-producing genera such as *Blautia*, *Bacterioides*, *Roseburia,* and *Coprococcus* [125]. A randomized trial showed that omega-3 PUFA supplementation induces a reversible increase in several SCFA-producing bacteria [126].

Since antibiotic administration represents an aggressive and non-selective means of manipulation of gut microbiota composition, its role in CRC management seems to be controversial. Although preclinical evidence showed that gut microbiome depletion seems to inhibit cancer progression [127], multiple lines of evidence highlight how antibiotics can undermine immunotherapy efficacy or promote disease progression emphasizing microbial dysbiosis [128,129].

Of course, a potential strategy of CRC prevention and management is represented by probiotics and fecal microbiota transplantation (FMT). Probiotics are living microorganisms with the potential to positively influence resident microbiota, intestinal epithelium cells, and the immune system, and they are generally considered safe and well tolerated in healthy subjects [130]. A randomized trial with *Lactobacillus* and *Bifidobacterium* strains significantly reduced the levels of proinflammatory cytokines such as TNF-α, IL-6, IL-10, IL-12, IL-17, and IL-22 and prevented post-surgical complications.

FMT consists of the transplantation of gut microbiota from healthy donors to patients to restore intestinal dysbiosis and reduce the activation of inflammatory, proliferative, and procarcinogenic pathways. These specimens are prepared according to well-established protocols to avoid potential risk factors such as viruses and parasites and stored in banks of donated feces [131]. Treatment with chemotherapy and ICIs can result in adverse events including colitis. FMT treatment has been shown to improve ICI-induced colitis in cancer patients [132]. Additionally, FMT reduced the severity of intestinal mucositis and diarrhea following FOLFOX treatment in preclinical models by suppressing IL-6 levels, increasing the number of goblet cells and zonula occludens-1, decreasing apoptotic and NFkB-positive cells as well as the expression of Toll-like receptors and MYD88, leading to a restoration of gut microbiota composition without complications such as bacteremia [133]. Another study conducted in a mouse model to assess the efficacy of FMT to reverse antibiotic- and chemotherapy-induced gut dysbiosis suggests that FMT may effectively help in preventing acute intestinal inflammation and mucosal barrier dysfunction. In particular, the administration of FMT reduced the proportions of pathogenic species and an increase of the relative distribution of Clostridium scindens and Faecalibacterium prausnitzii, which are species that exhibited anti-inflammatory properties [134].

Finally, as demonstrated by Hefazi et al. in cancer patients treated with cytotoxic chemotherapy, FMT treatment determined a reduction of multiply recurrent Clostridium difficile infection (CDI) and diarrhea episodes remarking its highly therapeutic efficacy [135].

## 4. Future Perspectives and Conclusions

Despite the recent advances in the systemic treatment of molecularly selected CRC patients with advanced disease (i.e., pembrolizumab in MSI [99] or the association of the anti-BRAF encorafenib, the anti-MEK binimetinib and the anti-EGFR cetuximab in BRAF V600E mutated [136] tumors), the survival benefit is limited to a small percentage (10–20%) of patients harboring these alterations.

The use of CTCs, ctDNA, miRNAs, and lncRNA could help find new potentially targetable biomarkers for the management of CRC. Furthermore, as a minimally invasive and repeatable procedure, liquid biopsy can improve CRC screening, early diagnosis, clinical staging, and prognostic stratification, allowing a higher rate of cure. Moreover, liquid biopsy might be useful to monitor minimal residual disease after surgical treatment, possibly allowing a finer modulation of the adjuvant systemic therapy, integrating clinico-pathological risk factors and ctDNA or CTC detection. Finally, if properly integrated with clinical and instrumental assessment, liquid biopsy might help monitor disease progression, treatment efficacy, and acquired resistance to chemotherapy and targeted agents in CRC.

Of course, there is urgent need to optimize pre-analytical and analytical processing for clinical validity, to standardize laboratory methods in ensuring the reproducibility of the results and to properly assess the cost-effectiveness [137]. Indeed, the lack of clinical applicability is currently due to the large quantity of liquid biopsy assays, with different detection limits, sensitivity, and specificity [138]. To solve the pitfalls for liquid biopsies due to the difficulty of CTC detection, the application of various microfluidic platforms based on CTC characteristics has been explored [139]. Recently, for the selection of CTC, a “negative depletion” microfluidic chip has been developed [140]. In this system, named leukapheresis, the leukocyte depletion strategy can enrich for untagged CTCs in a “tumor-independent” manner applicable to all tumor types, as demonstrated in several tumor types [141,142,143,144].

CTC analyses performed on leukapheresis products should improve the reach of liquid biopsies in metastatic cancer, and combined with CTC detection, they may play a critical role in screening high-risk patients for early cancer, identifying the tissue of origin, and reducing the need for invasive biopsies.

Therefore, once the multiple ongoing randomized phase II–III trials will define and validate the role of liquid biopsy especially in the adjuvant setting of early CRC (Table 1), a process of harmonization of procedures and data will be necessary to transfer from bench to bedside this important tool of personalized medicine.

On the other hand, it is clear that CRC carcinogenesis is also defined by gut microbiota metabolic activity and its dysbiotic composition. Therefore, the integrated analysis of the gut microbiome and its interactions with the host, anticancer drugs, and other exogenous factors [7,139] is essential to improve the outcomes of CRC patients. Recent findings support the potential of microbial markers in cancer diagnosis and prognosis and the potential of FMT or pre-probiotics in remodeling the tumor microenvironment or in potentiating antitumor immunity. Continuous monitoring of changes in microbiota profiles and biomarkers may help in the identification of dysplasia. In addition, in this context, an important collaborative effort is required to elucidate the role of the gut microbiota in modulating responses to cancer treatment, and this aspect is particularly clear in several ongoing clinical trials investigating the effect of FMT in patients with cancer who are refractory to ICI. These trials, along with further validations, will determine whether the selective modulation of gut microbiota, either by FMT, probiotic treatment, or other means, enables CRC patients to overcome resistance to chemotherapy or immunotherapy (Table 2). Of course, a more complete and holistic approach toward cancer treatment should include host–microbiota interactions as important screening and treatment factors.

## Figures and Tables

**Table 1:** List of major ongoing prospective trials investigating liquid biopsy in colorectal cancer.

Brief Study Title	NCT Number/Study Name	Country Study Period	Stage	N	Study Type/Phase/Endp	Target/Assay-Test (If Available)	Study Overview/Schematic Description
ctDNA Analysis Informing ACT in Stage III CRC	ACTRN 12617001566325 (DYNAMIC-III)	Australia 2017–2024	III	1000	Int rand/2–3/DFS, OS, ctDNA clear	ctDNA	Surgery followed by ctDNA detection and clinician’s choice ACT (no ACT > fluoropyrimidine > XELOX/FOLFOX) followed by randomization to: (1) SOC arm: SOC ACT (ctDNA blinded) or (2) EXP arm ctDNA+: escalated ACT (FOLFOXIRI allowed) or (3) EXP arm ctDNA-: de-escalated ACT
ctDNA Analysis Informing ACT in Stage II CRC	ACTRN 12615000381583 (DYNAMIC II)	Australia 2015–2024	II	450	Int rand/2/DFS, OS, ctDNA clear	ctDNA	Surgery followed by ctDNA detection and randomization to: (1) SOC arm: SOC, (2) EXP arm ctDNA+: ACT (fluoropyrimidine or XELOX/FOLFOX according to clinician’s choice), (3) EXP arm ctDNA-: follow-up
Tracking mutations in ctDNA to predict relapse in early CRC	NCT04050345 (TRACC)	UK 2016–2024	HR II-III	1000	Int rand/3/DFS, OS, QoL	ctDNA/RM NGS	Surgery followed by randomization to: (1) SOC arm: SOC ACT, (2) EXP arm: ctDNA guided de-escalated ACT—If ctDNA-: CAPOX × 3 months is reduced to cape x 6 months and CAPE × 6 months is reduced to observation—After 3 months if ctDNA+: switch to CAPOX
ctDNA Based Decision for ACT in CRC Stage II Evaluation	NCT04089631 (CIRCULATE)	Germany Austria Switzerland 2019–2026	II	4812	Int rand/3/DFS, OS, TT ctDNA clear	ctDNA/Dresden NGS	After radical surgery, ctDNA+ are randomized to: (1) CAPE × 6 months (or CAPOX × 3–6 months, investigator choice) or (2) follow-up and CtDNA- are randomized to: (1) follow-up inside the study or (2) follow-up outside the study (off-study)
Decision for ACT in stage II CRC based on ctDNA	NCT04120701 (CIRCULATE-PRODIGE 70)	France 2019–2026	II	1980	Int rand/3/DFS, TTR, OS, ctDNA clear	ctDNA/ddPCR of 2 methylated probes	After radical surgery, ctDNA+ are randomized to: (1) ACT (FOLFOX) or (2) surveillance inside the trial. CtDNA- are randomized to (1) surveillance inside the trial or (2) surveillance outside the trial
ctDNA guided ACT in stage II CRC according the trials within cohorts design	Planning NTR6455 (MEDOCC CrEATE)	Netherland 2018–2022	LR II	1320	Int rand/3/DFS, OS, QoL	ctDNA/gene panel (PG Dx elio platform)	Surgery followed by randomization to: (1) EXP arm: if ctDNA+ FOLFOX/CAPOX × 6 months or follow-up (patient choice), (2) EXP arm: if ctDNA- follow-up, (3) SOC arm: follow up (ctDNA tested later)
Intervention Trial Implementing Non-invasive ctDNA Analysis to Optimize the Operative and Postoperative Treatment for CRC Patients	NCT03748680 (IMPROVE-IT)	Denmark 2018–2025	I-II	64	Int rand/2/DFS, LR, TTR, OS, ctDNA clear	ctDNA/NGS + ddPCR	After radical surgery, ctDNA+ patients with no indication to ACT according to DCCG guidelines are randomized to: (1) SOC arm: intensified follow-up or (2) EXP arm: CAPOX or FOLFOX followed by intensified follow-up
ctDNA Testing in Predicting Treatment for Patients With Stage IIA CRC After Surgery	NCT04068103 (COBRA)	USA Canada 2019–2024	IIA	1408	Int rand/2–3/ctDNA clear, RFS, OS, TTR	ctDNA	After radical surgery, patients are randomized to: (1) SOC arm: active surveillance (blood stored and tested for ctDNA later), (2) EXP arm ctDNA+: FOLFOX or XELOX × 6 months, (3) EXP arm ctDNA-: active surveillance
Early identification and treatment of occult metastatic disease in stage III CRC	NCT03803553	USA 2020–2023	III	500	Int/3/DFS, OS, ctDNA clear	ctDNA	Surgery followed by SOC ACT followed by ctDNA assessment for MRD: (1) ctDNA+ MSI-h: Nivolumab, (2) ctDNA+ BRAF V600E: Enco/Bini/Cet × 6 months, (3) ctDNA+ others: randomization to FOLFIRI × 6 months or SOC observation, (4) ctDNA-: SOC observation
Post-surgical Liquid Biopsy-guided Treatment of Stage III and HR Stage II CRC Patients	NCT04259944 (PEGASUS)	Italy Spain 2020–2023	HR II- III	140	Int/2/DFS, OS, TT, QoL, ctDNA clear	ctDNA/LUNAR1 test	After surgical surgery: (1) ctDNA+: CAPOX × 3 months, (2) ctDNA-: CAPE × 6 months and early switch to CAPOX if ctDNA+ after first cycleAfter ACT: (3) ctDNA+/+: FOLFIRI × 6 months, 2 ctDNA-/+: CAPOX × 3 months, switch to FOLFIRI if still ctDNA-/+, 3ctDNA+/-: CAPE × 3 months, switch to FOLFIRI if ctDNA+/+ after 3 LB, (4) ctDNA-/-: follow-up, switch to CAPOX if ctDNA+ after 2 LB
Initial Attack on Latent Metastasis Using TAS-102 for ctDNA Identified CRC After Curative Resection	NCT04457297 (ALTAIR)	Japan 2020–2023	I-IV resected	240	Int rand/3/DFS, OS, TT	ctDNA/Signatera test	After radical surgery and SOC ACT, ctDNA+ patients are randomized to: (1) EXPrm: TAS-102 × 6 months or (2) SOC arm: placebo x 6 months
A Phase II Clinical Trial Comparing the Efficacy of RO7198457 Versus Watchful Waiting in Patients With ctDNA positive, Resected HR Stage II and Stage III CRC	NCT04486378 BNT122-01	USA 2020–2023	HR II-III	201	Int rand/2/DFS, OS, TTR, TTF, TT	ctDNA	After radicalsurgery, ctDNA+ patients receive SOC ACT and are then randomized to: (1) EXP arm: RO7198457 (a personalized cancer vaccine) or (2) SOC arm: watchful waiting
ctDNA Analysis to Optimize Treatment for Patients with CRC	NCT03637686 (IMPROVE)	Denmark 2018–2026	III	1800	Obs/DFS	ctDNA	Part I—Surgery: ctDNA detection pre- and postoperative. Part II—Surveillance: ctDNA detection over 5 years follow-up
BESPOKE study of ctDNA guided therapy in CRC	NCT04264702	USA 2020–2024	II-III	1000	Obs/DFS	ctDNA/Signatera test	To examine the impact of SIGNATERA test on ACT decisions and clinical outcomes during a 2-year follow-up
Use of ctDNA for Monitoring of Stage III CRC	NCT02842203 (PRO16020374)	USA 2016–2021	III	150	Obs/OS, PFS	ctDNA	ctDNA serial assessment up to 5 years and correlation with CEA and clinical outcomes
The implication of ctDNA in the recurrence surveillance of stage II and III CRC	NCT03416478 FFJC2017-01	China 2018–2020	II-III	50	Obs/DFS, OS	ctDNA	ctDNA serial assessment before and after curative surgery up to 2 years of follow-up
A phase II Clinical Trial comparing Efficacy of RO7198457 vs. watchful waiting in ctDNA positive stage II–III resected CRC	NCT04486378	USA 2020–2027	II-III	201	Int rand/2/DFS, RFS, TTR, TTF, OS, TT, ctDNA clear	ctDNA	To compare the efficacy of RO7198457 vs. watchful waiting after surgery and SOC ACT in ctDNA positive stage II–III CRC
ctDNA as a Prognostic Marker for Postoperative Relapse in Early and Intermediate Stage CRC	NCT03312374	China 2017–2020	II-III	350	Obs/DFS	ctDNA/NGS	ctDNA serial assessment before and after curative surgery and ACT up to 2 years of follow-up
The Implication of Plasma ctDNA Methylation Haplotypes in Detecting CRC and Adenomas	NCT03737591	China 2018–2020	I-IV adenomas healthy	500	Obs	ctDNA/NGS	To evaluate the sensitivity and specificity of ctDNA methylation haplotypes in detecting CRC and adenomas
Dynamic monitoring of ctDNA methylation to predict relapse in stage II–III CRC after radical resection	NCT03737539	China 2018–2022	II-III	300	Obs/DFS	ctDNA/NGS	To correlate and compare postoperative, pre- and post-ACT ctDNA methylation markers with radiological imaging and clinical outcomes
Ct-DNA Testing in Guiding Treatment for Patients With Advanced or Metastatic CRC	NCT03844620	USA 2019–2020	III (uncurable)-IV	100	Int/2/TT, ctDNA clean, QoL, ORR, OS	ctDNA	Monitoring and correlating ctDNA changes and radiological progression or TT incidence during third-line SOC (TAS-102/Regorafenib) (arm A) vs. third-line SOC alone (arm B)
Predictive and Prognostic Value of Inflammatory Markers and microRNA in Stage IV CRC	NCT04149613	USA 2018–2021	IV	100	Obs	miRNAs	To evaluate the expression of selected microRNAs and inflammatory markers in patients with stage IV CRC and assess their correlation with tumor location, dietary patterns, survival rates, response to systemic chemotherapy, and other clinic-pathological parameters
Molecular Pathology of CRC: Investigating the Role of Novel Molecular Profiles, microRNAs, and their Targets in CRC Progression	NCT03309722	UK 2008–2025	I-IV	1000	Obs/OS, DFS, LR, DR	miRNA	Single-center observational cohort study of prospectively recruited patients for biomarker evaluation and identification of novel biomarkers
Contents of Circulating Extracellular Vesicles: Biomarkers in CRC Patients	NCT04523389 (ExoColon)	France 2020–2021	I-IV	172	Obs/OS, PFS, LR, DR	Exosomes	To investigate the prognostic and predictive role of exosomes and their contents (miRNAs and others)
ColoCare Transdiciplinary Research in CRC Prognosis	NCT02328677	USA 2007–2030	I-IV	5000	Obs/OS, DFS, QoL, TT	ctDNA, MiRNAs	To investigate the prognostic and predictive role of liquid biopsy in CRC patients in a 5-year follow-up
Timing To Minimally Invasive Surgery After Neoadjuvant Chemoradiotherapy For Rectal Cancer: A Multicenter Randomized Controlled Trial—Biomarkers SubStudy	NCT03962088 (TiMiSNAR)	Italy 2019–2023	II-III	200	Obs/pCR, DFS	miRNA/miRNeasy Mini kit by Qiagen	(1) To investigate the association between pre-neoadjuvant and post-neoadjuvant expression levels of miRNA with pCR. (2) To investigate the correlation between changes in expression levels of miRNA following complete surgical resection with DFS and the relation between changes in miRNA during surveillance and tumor relapse
microRNAs Tool for Stratifying Stage II CRC: a Perspective Study of ACT	NCT02635087	China 2015–2025	II	630	Obs/DFS, OS	miRNA	To investigate the predictive and prognostic role of miRNAs in stage II CRC, stratifying patients at “high risk” and at “low risk” of recurrence according to a six miRNAs tool
Assessment Of Long Noncoding RNA CCAT1 Using Real-Time Polymerase Chain Reaction In CRC patients	NCT04269746	Egypt 2020–2021	Diagnostic	100	Obs	lncRNA	To evaluate the clinical utility of detecting long non-coding RNA (CCAT1) expression in diagnosis of CRC patients and its relation to tumor staging

ctDNA: circulating tumor DNA; Endp: study endpoints if not reported in study description; N: planned enrollment; Obs: observational; Int: interventional; CT: chemotherapy; LB: liquid biopsy; dPCR: digital PCR; ddPCR: digital droplet PCR; SOC: standard of care; ACT: adjuvant chemotherapy; NA: not available; Enco/Bini/Cet: Encorafenib/Binimetinib/Cetuximab; HR: high risk according to histopathological factors; LR: low risk according to hystopatological factors; ctDNA clear: ctDNA clearance or modification rate of every study arm and correlation with clinical outcome measures (according to the design of each study); OS: overall survival; DFS: disease-free survival; RFS: relapse-free survival; LR: local recurrence; DR: distant recurrence; QoL: quality of life; TT: treatment toxicity/treatment-related adverse events; pCR: pathologic complete response; TTF: time to treatment failure; TTR: time to recurrence.

**Table 2:** List of major ongoing prospective trials investigating the role of microbiota in colorectal cancer.

Brief Study Title	NCT Number/Study Name	Country Study Period	Stage	N	Study Type/Phase (If Applicable)	Intervention	Study Overview/Schematic Description
Gut Microbiome Dynamics in Metastasized or Irresectable CRC	NCT03941080 GIMICC	Netherlands 2020–2022	IV	300	Obs	Fecal and blood sample collection + behavioral questionnaire at baseline and every 3 months	To investigate characteristics and alterations of the gut microbiome and its predictive value for RR and TT during CT for mCRC
Gut Microbiome and Oral Fluoropyrimidine Study in Patients With CRC	NCT04054908 GO	USA 2018–2022	all	60	Obs	1 stool sample at baseline and at least 1 stool sample during treatment + questionnaires regarding bowel habits and dietary habits	To investigate the alterations of the gut microbiome occurring in three cohorts of CRC: Cohort A: patients treated with CAPE as SOC, Cohort B: patients treated with TAS-102 with or without Y-90 radioembolization in T, Cohort C: patients treated with CAPE + pembrolizumab + bevacizumab in T
Human Intestinal Microbiome and Surgical Outcomes in Patients Undergoing CRCCancer Surgery	NCT04005118 Microbiota	France	all	50	Obs	2 fresh fecal samples for LM detection (1 pre- and 1 post-operatively) + 1 intraoperatively sample for MAM	To investigate the association between microbiome composition and occurrence of postoperative complications (anastomotic leakage, surgical site infection, prolonged postoperative ileus)
Bowel Preparation Impact on the Intestinal Microbiome: Oral Preparation vs. Enema	NCT04013841 BowelPrepMicrobiome	USA 2020–2022	Left-sided CRC	60	Int rand	Stool samples before and after bowel preparation and surgery	To investigate differences in microbiome composition according to oral and enema bowel preparation for left side colon surgery and its correlation with surgical outcomes
The Role of Microbiome in Cancer Therapy	NCT02960282	USA 2016–2021	IV	80	Obs	Fecal specimen collection at baseline, prior to each cycle and at PD or off-treatment	To investigate microbiome composition, its gene and protein expression profile and correlation with RR and TT in two cohorts: Cohort A: patients treated with FOLFOX or FOLFIRI CT backbone as first line regimen, Cohort B: patients treated with pembrolizumab
Colorectal Cancer Cohort Study	NCT04185779 COLO-COHORT	UK 2019–2024	diagnostic	15000	Obs	Blood and fecal tests + behavioral questionnaires	To develop a prediction model to stratify patients at risk of having adenomas or CRC (past medical history, family history, blood tests, FIT level, colonscopy, and microbiome stool)
Stool and Blood Sample Bank for CRC Patients	NCT04638751 ARGONAUT	USA 2020–2024	III–IV	4000		2 blood and stool samples each over a 6-month period	To determine whether the microbiome composition can predict PFS and OS in different cohorts of cancer patients (NSCLC, CRC, TNBC, and PC) treated with CT or IT. To identify correlations between microbiome composition and immune markers
Omega-3 Fatty Acid for the Immune Modulation of Colorectal Cancer	NCT03661047 OMICC	USA 2019–2023	I-III, HR adenomas	36	Int rand/2/	Blood and stool samples + lifestyle questionnaire + nutritional survey between CRC/adenomas detection and surgery	To evaluate the effect of a 30-day administration of AMR101 (VASCEPA, icosapent ethyl) on MO3PUFA composition, gut microbiome, and immune system elements concentration (CD8+ T cells, CD49b, CTLA-4, PD-L1, PD-1, LAG-3, IL10, FOXP3) in both normal and tumor tissue
Metagenomic Evaluation of the Gut Microbiome in Patients With Lynch Syndrome and Other Hereditary Colonic Polyposis Syndromes	NCT02371135	USA 2015–2021	High hereditary CRC risk	225	Obs	Stool sample + Brief Diet and Lifestyle Questionnaire before every colonscopy	To investigate the association of the gut microbiome and dietary factors with risk of adenoma or cancer in Lynch syndrome and other hereditary colonic polyposis syndrome patients
Pilot Trial of Resistant Starch in Stage I-III CRC Survivors	NCT03781778	USA 2018–2020	I–III	24	Int rand/2	Stool samples + Diet questionnaire at beginning and at 8 weeks	To compare the effect of a 8-week consumption of foods made of resistant (experimental arm) or corn (control arm) starch in addition to usual daily diet in modifying markers of inflammation, insulin resistance, and gut microbiome composition of CRC patients
Development and Analysis of a Stool Bank for Cancer Patients	NCT04291755	USA 2019–2021	all	100	Obs	Five stool, blood, and urine samples each over a 12-month period	To investigate the impact of gut microbiota on the efficacy of immune checkpoint inhibitors in NSCLC and CRC patients
Microbiome and Rectal Cancer	NCT04223102	USA 2020–2027	II–III	40	Int	Serial rectal biopsy specimens in a 5-year follow-up	To investigate the association between microbiome and pathologic response to neoadjuvant therapy in rectal cancer
Fecal Microbiota Transplant (FMT) Capsule for Improving the Efficacy of Anti- PD-1	NCT04130763	China 2020–2021	IV	10	Int	Induction dose with FMT capsules one week before anti-PD-1 treatment beginning followed by maintenance dose	To determine whether the FMT capsule improves ORR of anti-PD-1 treatment in resistant/refractory gastrointestinal cancer patients

Obs: observational; RR: response rate; TT: treatment toxicity/treatment-related adverse events; SOC: standard of care; T: clinical trial; LM: luminal microbiota; MAM: mucosal associated microbiota; CT: chemotherapy; IT: immunotherapy; NSCLC: non-small cell lung cancer; TNBC: triple negative breast cancer; PC: pancreatic cancer; HR: high-risk; MO3UFA: marine omega-3 polyunsaturated fatty acid; FMT: fecal microbiota transplant.

## Abbreviations

5-FU	5-Fluorouracil
ACT	Adjuvant Chemotherapy
Akt	Protein Kinase B
APC	Adenomatous Polyposis Coli
APCMin	Multiple Intestinal Neoplasia, a mutant allele of the Murine Adenomatous Polyposis Coli Locus
ATM	Ataxia Telangiectasia Mutated Serine/Threonine Kinase
AUC	Area Under the Curve
BIRC3	Baculoviral IAP Repeat Containing 3
BMP	Bone Morphogenetic Protein
CA19-9	Carbohydrate Antigen 19-9
cAMP	3′-5′-Cyclic Adenosine Monophosphate
CDI	Clostridium Difficile Infection
CEA	Carcino Embryonic Antigen
CEA	Carcinoembryonic Antigen
cf-DNA	Cell-Free DNA
CIMP	CpG Island Methylator Phenotype
CPSF4	Cleavage And Polyadenylation Specific Factor 4
CRC	Colorectal Cancer
CSC	Cancer Stem Cells
CT	Computed Tomography
CT	Chemotherapy
CTCs	Circulating Tumor Cells
ctDNA	Circulating Tumor DNA
ctDNA clear	ctDNA clearance or modification rate of every study arm and correlation with clinical outcome measures (according to the design of each study)
ctRNAs	Circulating Tumor RNAs
Cxcl2	C-X-C Motif Chemokine Ligand 2
DCE	Dynamic Contrast-Enhanced
ddPCR	Digital Droplet PCR
DDR	DNA Damage Response
DFS	Disease-free Survival
dPCR	Digital PCR
DR	Distant Recurrence
EGFR	Epidermal Growth Factor Receptor
EMT	Epithelial–Mesenchymal Transition
Enco/Bini/Cet	Encorafenib/Binimetinib/Cetuximab
Endp	Study Endpoints if not Reported in Study Description
ETBF	Enterotoxigenic Bacteroides Fragilis
EVs	Extracellular Vesicles
FMT	Fecal Microbiota Transplantation
Fn	*Fusobacterium Nucleatum*
FOBT	Fecal Occult Blood Test
FOXO	Forkhead Box O3
GC	Gastric Cancer
GI	Gastrointestinal
Gli1	Glioma-Associated Oncogene Homolog 1
GLOBOCAN	Global Cancer Statistics
GMMs	Metabolites of the Intestinal Microbiome
HIF-1	Hypoxia-Inducible Factor 1
HR	Hazard Ratio
HR	High-Risk According to Histopathological Factors
HR	High-Risk
IAPs	Inhibitor of Apoptosis Proteins
ICIs	Immune Checkpoint Inhibitors
IECs	Ileal Intestinal Epithelial Cells
IL-17	Interleukin-17
Int	Interventional
IT	Immunotherapy
LB	Liquid Biopsy
LM	Luminal Microbiota
lncRNAs	Long non-coding RNAs
LR	Low-Risk According to Histopatological Factors
LR	Local Recurrence
MAM	Mucosal Associated Microbiota
mCRC	Metastatic CRC
MDM2	Mouse Double Minute 2
MEK	Mitogen-Activated Protein Kinase
MIR	MicroRNA
miRNAs	MicroRNAs
MO3UFA	Marine Omega-3 Polyunsaturated Fatty Acid
mRCC	Metastatic Renal Cell Carcinoma
MRD	Minimal Residual Disease
MRI	Magnetic Resonance Imaging
mSEPT9	methylated Septin 9
MSI	Micro-Satellite Instability
mTOR	Mammalian Target of Rapamycin
MYD88	Myeloid Differentiation Primary Response 88
N	Planned Enrollment
NA	Not Available
ncRNAs	non-coding RNAs
NEAT1	Nuclear Enriched Abundant Transcript 1
NFkB	Nuclear Factor Kappa-Light-Chain-Enhancer of Activated B Cells
NSCLC	Non-Small Cell Lung Cancer
NTBF	Non-Enterotoxigenic Bacteroides Fragilis
Obs	Observational
OMVs	Outer Membrane Vesicles
OS	Overall Survival
PACs	Proanthocyanidins
PC	Pancreatic Cancer
pCR	Pathologic Complete Response
PD-1	Programmed Death-1
PFS	Progression-Free Survival
PI3K	Phosphatidylinositol 3-Kinase
PLGC	Precancerous lesions of gastric carcinoma
PTEN	Phosphatase and Tensin Homolog
PUFAs	Omega-3 Polyunsatured Fatty Acids
PVAs	Hydroxy-Phenylvaleric Acids
PVLs	Hydroxy-Phenyl-γ-Valerolactones
QoL	Quality of Life
RECIST	Response Evaluation Criteria in Solid Tumors
RFS	Relapse-Free Survival
ROC	Receiver Operating Characteristic Curve
ROS	Reactive Oxygen Species
RR	Response Rate
SCFA	Short-Chain Fatty Acid
SMAD4	Mothers Against Decapentaplegic Homolog 4
SOC	Standard of Care
T	Clinical Trial
TDEs	Tumor-Derived Exosomes
TFH	Follicular T-helper Cell
TGF-β	Transforming Growth Factor Beta
TLR4	Toll-like Receptor 4
TNBC	Triple Negative Breast Cancer
TNF-α	Tumor Necrosis Factor- alpha
TNM	Classification of Malignant Tumors (Tumor-Nodes-Metastasis)
TNMB	Tumor-Nodes-Metastasis-Blood
TT	Treatment Toxicity/Treatment-Related Adverse Events
TTF	Time to Treatment Failure
TTR	Time to Recurrence
VEGF	Vascular Endothelial Growth Factor
